# *β-catenin* Overexpression in the Nucleus Predicts Progress Disease and Unfavourable Survival in Colorectal Cancer: A Meta-Analysis

**DOI:** 10.1371/journal.pone.0063854

**Published:** 2013-05-24

**Authors:** Zhigang Chen, Xin He, Minyue Jia, Yang Liu, Dihong Qu, Dang Wu, Pin Wu, Chao Ni, Zhigang Zhang, Jun Ye, Jinghong Xu, Jian Huang

**Affiliations:** 1 Department of Oncology, Second Affiliated Hospital, Zhejiang University School of Medicine, Hangzhou, China; 2 Cancer Institute (Key Laboratory of Cancer Prevention & Intervention, National Ministry of Education, Provincial Key Laboratory of Molecular Biology in Medical Sciences), Zhejiang University School of Medicine, Hangzhou, China; 3 Department of Hematology, Second Affiliated Hospital, Zhejiang University School of Medicine, Hangzhou, China; 4 Department of Endocrinology, Second Affiliated Hospital, Zhejiang University School of Medicine, Hangzhou, China; 5 Department of Pathology, Second Affiliated Hospital, Zhejiang University School of Medicine, Hangzhou, China; The Chinese University of Hong Kong, Hong Kong

## Abstract

**Background:**

*β-catenin* plays a key role in the progression of colorectal cancer (CRC). However, its prognostic significance for patients with CRC remains controversial.

**Methodology:**

Identical search strategies were used to search relevant literatures in the PubMed, Embase and Web of Science databases. The correlation between *β-catenin* expression and clinicopathological features and prognosis was analyzed.

**Principal Findings:**

A total of 18 studies met the inclusion criteria, which comprised 3665 cases. Meta-analysis suggested that *β-catenin* overexpression in the nucleus was significantly associated with disease free survival (DFS) (n = 541 in 3 studies; HR = 1.87, 95% CI: 1.28–2.71; Z = 3.26; P = 0.001) and overall survival (OS) for CRC patients (n = 2630 in 10 studies; HR = 1.55, 95% CI: 1.12–2.14; Z = 2.62; P = 0.009). However, there was no significant association between *β-catenin* expression in the cytoplasm and OS (n = 1327 in 3 studies; HR = 1.04, 95% CI: 0.88–1.24, Z = 0.46, P = 0.643). The combined odds ratio (OR) of *β-catenin* in the nucleus indicated that *β-catenin* overexpression was associated with advanced stage CRC (n = 950 in 7 studies; OR = 0.71, 95% CI: 0.53–0.94; Z = 2.35; P = 0.019) and metastasis of CRC (n = 628 in 5 studies; OR = 0.49, 95% CI: 0.25–0.96, Z = 2.06, P = 0.039). *β-catenin* overexpression in the nucleus had no correlation with the tumor site (colon or rectum), differentiation grade, lymph node status or depth of invasion. The pooled ORs were 1.09 (95% CI: 0.41–2.91, Z = 0.18, P = 0.856), 1.27(95% CI: 0.76–2.10, Z = 0.92, P = 0.357), 0.71(95% CI: 0.46–1.09, Z = 1.58, P = 0.115) and 0.82(95% CI: 0.4–1.68, Z = 0.53, P = 0.594).

**Conclusions:**

This study showed that *β-catenin* overexpression in the nucleus, rather than in the cytoplasm, appeared to be associated with progress disease and a worse prognosis for CRC patients.

## Introduction

Colorectal cancer (CRC) is the third most common human malignancy and the second highest cause of cancer-related death worldwide [1]. Despite the tremendous progress in treatment, the overall mortality of CRC is still approximately 40% [2]. Surgery is the fundamental treatment, but 30% to 50% of patients with stage II to III tumors relapse within 5 years following treatment [3]. Additionally, 5-FU or oxaliplatin, the most widely used anticancer agent, has become ineffective against CRC due to the development of intrinsic or acquired drug resistance. Therefore, it is important to uncover the biological mechanisms underlying the progression of the disease and develop strategies to intervene in this process.

Increasing evidence suggests that the existence of a small subset of bulk cancer cells, termed cancer initiating cells (CICs), are responsible for tumor progression, therapy resistance and disease relapse. Self-renewal and multilineage differentiation potential are two main traits of CICs. Since O’Brien and Ricci-Vitiani identified a CD133 positive subtype as colorectal cancer initiating cells (CCICs) in 2007, the molecular mechanisms sustaining CCICs have been slowly understood [4], [5]. The Wnt signaling pathway is one of the best-studied signaling cascades, and it has been suggested that this pathway plays an important role in maintaining the stemness and self-renewal capacity of CCICs [6]–[8]. Regulation of this pathway is realized through the level of *β-catenin* protein in the nucleus. *β-catenin* maintains a low cytoplasmic concentration through the destruction complex when the Wnt signaling pathway is unactivated. Otherwise, the destruction complex is dissolved and *β-catenin* accumulates in the cell and undergoes translocation to the nucleus, where it activates the expression of target genes, such as *CyclinD1, c-Myc, CD44* and *Survivin*, in conjunction with the TcF/LEF transcription molecule family. Thus, the level of *β-catenin* in the nucleus is an indicator of an active Wnt signaling pathway or CCICs [9]. *β-catenin* in the nucleus is expected to be a useful biomarker associated with disease progression and poor prognosis in CRC. Moreover, some researchers suggest that the accumulation of cytoplasmic *β-catenin* serves as a predictor of metastasis of CRC [10]. However, the correlations between the expression of *β-catenin* detected by immunohistochemistry and patient survival are highly variable and contradictory. Therefore, it is necessary to analyze the data on *β-catenin* and CRC to draw a reasonable conclusion about its prognostic significance.

In this study, we conducted a meta-analysis to investigate *β-catenin* expression and the prognosis of corresponding patients. The results showed that overexpression of *β-catenin* in the nucleus, rather than in the cytoplasm, was associated with progress disease and worse prognosis. The meta-analysis suggested that postoperative detection of *β-catenin* expression in CRC would help us develop better therapy strategies, distinguish high risk populations from the patients undergoing surgery and make better follow-up plans.

## Methodology

### Literature Search

We carried out a search of the PubMed, Embase and Web of Science databases using the following terms and all possible combinations: “*β-catenin,*” “Axin Signaling Complex,” “Wnt Signaling Pathway,” “Colorectal Neoplasms,” “Colorectal Cancer” and “prognosis.” The citation lists associated with all the studies were used to identify additional eligible studies. The reviews and bibliographies were also manually inspected to find related articles.

### Inclusion and Exclusion Criteria

The search results were included in our meta-analysis if they met the following inclusion criteria: (1) *β-catenin* expression evaluated in the human CRC tissues; (2) evaluation of the relationships between *β-catenin* expression and CRC pathological features or prognosis; (3) *β-catenin* expression examined by immunohistochemistry; (4) English language publications; and (5) sufficient information provided to estimate the hazard ratio (HR) or odds ratio (OR) and their 95% confidence intervals (CIs).

The following articles were excluded: (1) letters, case reports, reviews, and conference abstracts without original data; (2) non-English language articles; (3) articles from which the relevant data could not be extracted; and (4) overlapping articles or ones with duplicate data.

### Data Extraction and Assessment of Study Quality

All data were extracted independently by two authors (HX and JMY). For each study, the following characteristics were extracted: first author’s name, publication date, number of patients, gender of patients, tumor site, tumor stage, research technique used, antibody source, definition of *β-catenin* positive, relationship between *β-catenin* and survival and adjuvant therapy condition of patients. Controversial problems were arbitrated by the third investigator (XJH). Study quality was assessed independently by two investigators (HX and JMY) according to the Newcastle–Ottawa quality assessment scale [11].

### Statistical Analysis

We combined the data on *β-catenin* expression and pathological features into single categories: T1 and T2 stages, T3 and T4 stages, and well and moderate differentiation. ORs with 95% CIs were used to evaluate the association between *β-catenin* expression and clinicopathological factors, such as differentiation grade, Dukes’ stages, depth of invasion, lymph node status and metastasis. Survival data were extracted or calculated according to the methods described by Parmar [12]. Kaplan-Meier curves were read by Engauge Digitizer version4.1 (http://digitizer.sourceforge.net/). HR and its variance were used to estimate the impact of *β-catenin* expression on OS and DFS. Heterogeneity across studies was evaluated using a Chi-square-based Q statistical test [13]. The I^2^ statistic was also calculated to quantify the proportion of the total variation due to study heterogeneity [14]. A P>0.10 for the Q-test indicated a lack of heterogeneity among the studies. For studies with P>0.10, the pooled OR and HR estimates of each study were calculated by the fixed-effects model (the Mantel-Haenszel method). For studies with P≤0.10, the random-effects model (the DerSimonian and Laird method) was used [15], [16]. Egger’s test was used to examine the potential risk of publication bias. Publication bias was indicated when the P value from Egger’s test was <0.05. The statistical analyses were performed using STATA version 12.0 software (Stata Corporation, Collage Station, Texas, USA). All the P values were used for a two-sided test with significance at P<0.05.

## Results

### Description of Studies

**Figure 1:**
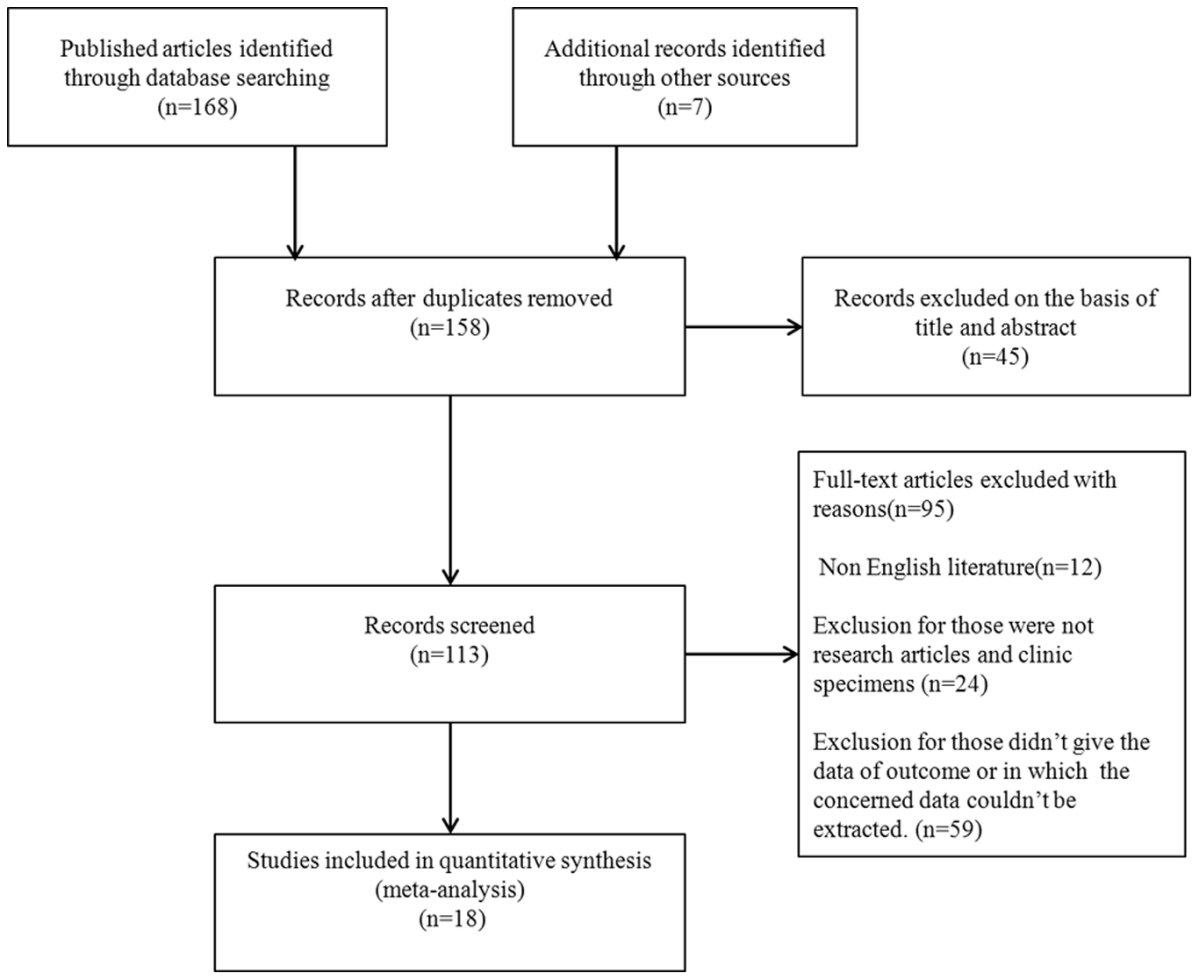
Flow diagram of study selection procedure.

We found 168 studies potentially eligible for inclusion based on the title (**Figure 1**). After scrutinizing the abstracts and full-text of these studies, 18 studies were ultimately chosen for this meta-analysis [10], [17]–[33]. Their characteristics are summarized in **Table 1**. The studies in our meta-analysis were published between 2000 and 2012. A total of 3703 CRC patients were enrolled and had the relationship between *β-catenin* expression and pathological features or disease free survival (DFS)/overall survival (OS) investigated. Immunohistochemistry was used to detect *β-catenin* expression in all the publications, but the sources of primary antibodies came from different companies.

**Table 1:** Characteristics of studies included in the meta-analysis.

First author	Year	Patient(M/F)	Country	Antibody source	Definition of *β-catenin* positive	HR estimation	Adjuvant therapy	Quality score
Andras	2012	100(52/48)	Hungary	Transduction Laboratories	>10%	HR for OS	Yes	8
Toth	2011	79(40/39)	Hungary	Transduction Laboratories	>10%	NA	Yes	7
Sun	2011	67(43/24)	China	Santa Cruz	>10%	NA	NA	8
Stanczak	2011	66(44/22)	Poland	DAKO	>10%	HR for OS	Yes	6
Ozguven	2011	60(38/22)	Turkey	Immunovision	>0%	NA	Yes	5
Morikawa	2011	955(381/574)	America	BD	Moderate/strong expression	HR for OS	NA	8
Matsuoka	2011	156(99/57)	Japan	Zymed Laboratories	>20%	HR for DFS	Yes	7
Pancione	2010	141(90/51)	Italy	BD	Weak/strong expression	HR for OS	Yes	7
Magnusson	2009	312(194/118)	Sweden	Transduction Laboratories	Moderate/strong expression	HR for OS	Yes	8
Pancione	2009	72(44/28)	Italy	BD	Weak/strong expression	HR for OS	Yes	7
Togo	2008	183(115/68)	America	NA	Moderate/strong expression	HR for DFS	NA	5
Chen	2008	60(29/31)	China	Beijing Zhongshan GoldenBridge	>10% of tumor cells	Survival curves for OS	NA	8
Martensson	2007	67(39/28)	Sweden	Sigma	>5%	Survival curves for OS	NA	7
Bravou	2005	125(NA)	Greece	DAKO	>10%	NA	NA	7
Fernebro	2004	269(173/96)	Sweden	Transduction Laboratories	Weak/strong expression	NA	Yes	5
Ougolkov	2002	202(110/92)	Japan	Transduction Laboratories	>10%	HR for OS and DFS	NA	6
Gina	2001	655(NA)	America	Transduction Laboratories	Moderate/strong expression	HR for OS	NA	5
Maruyama	2000	96(NA)	Japan	Transduction Laboratories	>10%	NA	NA	6

NA, not available; HR, hazard ratio; OS, overall survival; DFS, disease free survival.

### Methodological Quality of the Studies

Each of the 18 eligible studies included in our meta-analysis was assessed for quality according to the Newcastle–Ottawa Scale (NOS). NOS assessed eight items of methodology, which were categorized into the three dimensions of selection, comparability, and outcome. A maximum score of 1 was awarded for each item with the exception of the item related to comparability that allowed for scores of 2. For quality, scores ranged from 0 (lowest) to 9 (highest), and studies with scores of 6 or more were rated as high quality. Fourteen of the included studies obtained scores of 6 or more in methodological assessment, indicating that they were of high quality (**Table 1**).

### Impact of *β-catenin* Expression on Overall Survival and Disease-free Survival of Colorectal Cancer

**Figure 2:**
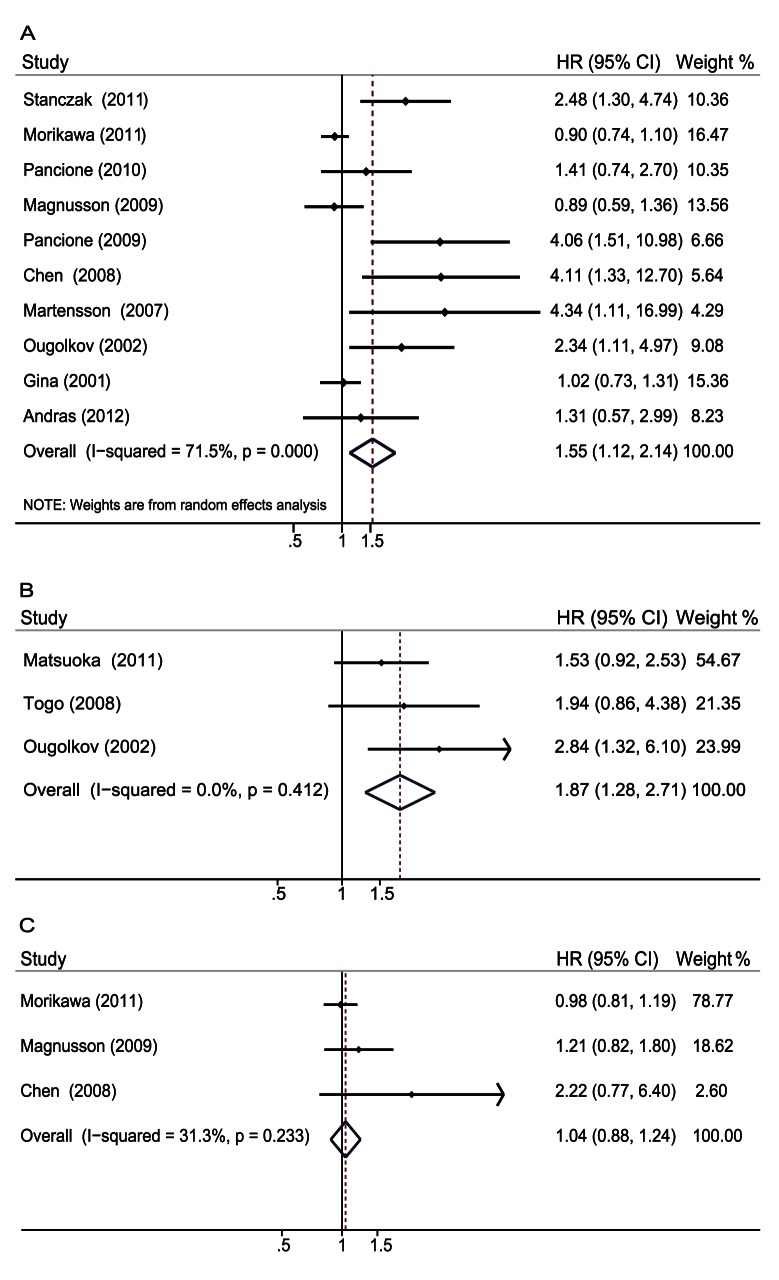
Forrest plot of hazard ratio for the association of *β-catenin* expression and survival. A. HRs with corresponding 95% CIs of the *β-catenin* expression in the nucleus with OS. B. HRs with corresponding 95% CIs of the *β-catenin* expression in the nucleus with DFS. C. HRs with corresponding 95% CIs of the *β-catenin* expression in the cytoplasm with OS. This showed that *β-catenin* expression in the nucleus, rather than in the cytoplasm, was associated with unfavorable prognosis of CRC patients.

The meta-analysis was performed on ten studies assessing the association of *β-catenin* expression in the nucleus with OS. The pooled HR was 1.55 (95% CI: 1.12–2.14; Z = 2.62; P = 0.009) (**Figure 2A**) with heterogeneity (I^2^ 71.5% P = 0.000). Three studies assessed the association of β-catenin expression in the nucleus with DFS; the pooled HR was 1.87 (95% CI: 1.28–2.71; Z = 3.26; P = 0.001) (**Figure 2B**) without heterogeneity (I^2^ 0% P = 0.412). These results suggested that *β-catenin* overexpression in the nucleus was significantly correlated with a worse prognosis of CRC and that *β-catenin* overexpression in the nucleus was an independent prognostic factor in CRC. We assessed three eligible studies and found that there was no significant association between *β-catenin* overexpression in the cytoplasm with OS; the combined HR was 1.04 (95% CI: 0.88–1.24, Z = 0.46, P = 0.643) without heterogeneity (I^2^ 31.3% P = 0.233) (**Figure 2C**). These studies indicated that *β-catenin* overexpression in the cytoplasm had no relationship with prognosis of CRC.

**Figure 3:**
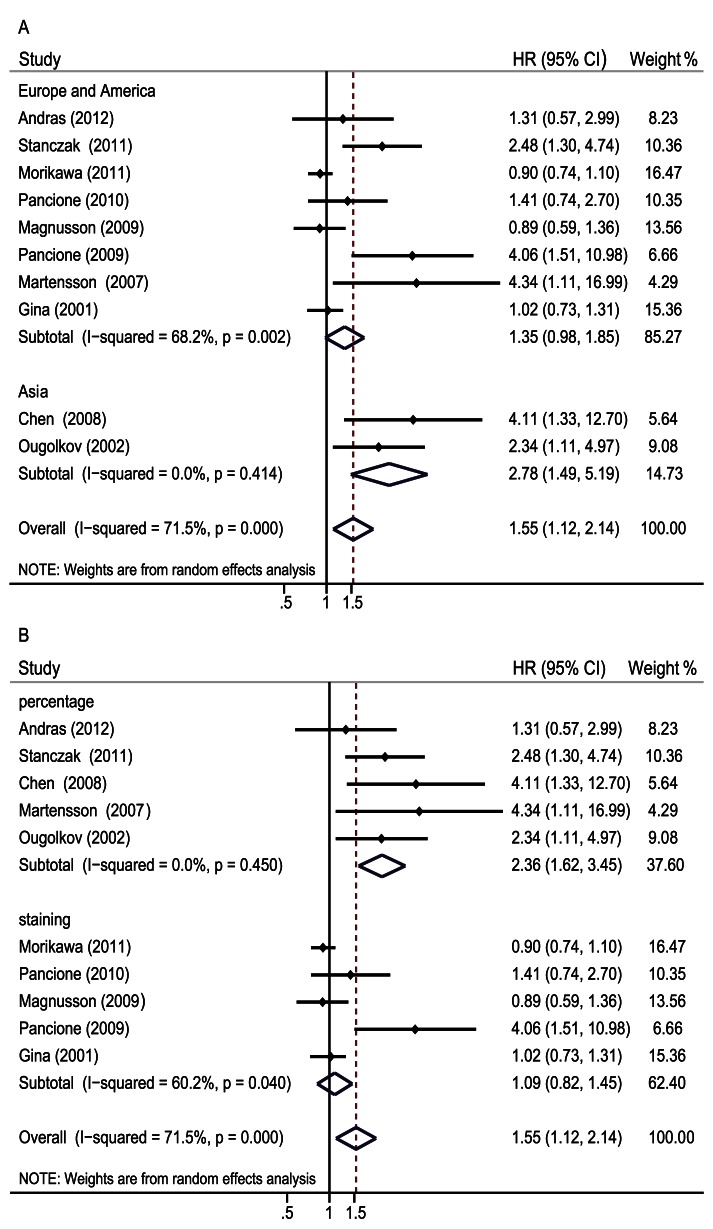
Forrest plot of hazard ratio for the association of *β-catenin* expression in the nucleus with overall survival by subgroup analysis. A. Subgroup analysis was performed by study location B. Subgroup analysis was performed by evaluation standards.

To explain the heterogeneity in OS, subgroup analysis was performed by the study location, source of primary antibodies, definition of *β-catenin* positive and adjuvant therapy condition. The results indicated that a significant relationship between *β-catenin* expression in the nucleus and OS was exhibited in Asian countries (HR 2.78, 95% CI: 1.49–5.19, Z = 3.21, P = 0.001) without heterogeneity (I^2^ 0% P = 0.414) (**Figure 3A**). Additionally, heterogeneity was not detected (I^2^ 0% P = 0.45) when the definition of *β-catenin* positive was a percentage (HR 2.36, 95% CI: 1.62–3.45, Z = 4.44, P = 0.000) (**Figure 3B**). When the analysis of OS was limited to studies with primary antibodies from the same company and with adjuvant therapy, heterogeneity still existed (I^2^ 79.6% P = 0.008 and I^2^ 56.4% P = 0.076). It indicated that the differences of patient ethnicity and evaluation standards contributed to heterogeneity in the results.

Taken together, these results suggested that *β-catenin* overexpression in the nucleus, rather than in the cytoplasm, influenced survival of CRC patients.

### Correlation of *β-catenin* Expression with Clinicopathological Parameters

**Figure 4:**
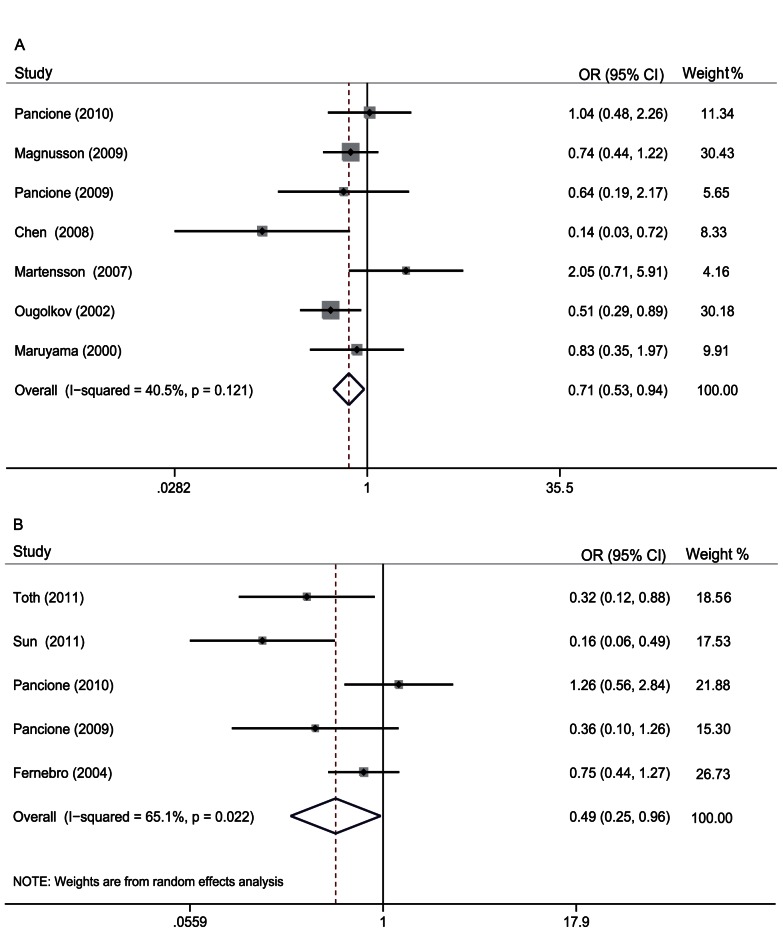
Forrest plot of odds ratios for the association of *β-catenin* expression in the nucleus with clinicopathological features. A. ORs with corresponding 95% CIs of the *β-catenin* expression in the nucleus with Dukes’ stages. OR<1 suggested that *β-catenin* in the nucleus was less in patients with Duke A/B than with Duke C/D and it was associated with advanced stage CRC. B. ORs with corresponding 95% CIs of the *β-catenin* expression in the nucleus with metastasis. OR<1 suggested that *β-catenin* in the nucleus was positively associated with metastasis of CRC.

Seven studies evaluated the correlation of *β-catenin* expression in the nucleus with Dukes’ stages. The pooled OR was 0.71 (95% CI: 0.53–0.94, Z = 2.35, P = 0.019) (**Figure 4A**) without heterogeneity (I^2^ 40.5% P = 0.121). This result suggested that *β-catenin* overexpression in the nucleus was associated with the progression of CRC. Five studies assessed the correlation of *β-catenin* overexpression in the nucleus with metastasis. The pooled OR was 0.49 (95% CI: 0.25–0.96, Z = 2.06, P = 0.039), indicating that *β-catenin* overexpression in the nucleus was associated with metastasis of CRC (**Figure 4B**). We also found that *β-catenin* overexpression in the nucleus had no relation with the tumor site (colon or rectum), differentiation grade, lymph node status or depth of invasion. The pooled ORs were 1.09 (95% CI: 0.41–2.91, Z = 0.18, P = 0.856), 1.27 (95% CI: 0.76–2.10, Z = 0.92, P = 0.357), 0.71(95% CI: 0.46–1.09, Z = 1.58, P = 0.115) and 0.82 (95% CI: 0.4–1.68, Z = 0.53, P = 0.594) **(** **Table 2** **)**.

**Table 2:** Meta-analysis of *β-catenin* expression in the nucleus.

Clinicopathological features	N	Cases	Analytical model	OR	95% CI	P valuefor OR	P value for heterogeneity
Differentiation grade	11	2584	REM	1.268	0.765–2.102	0.357	0.045
Duke stage	7	950	FEM	0.711	0.535–0.945	0.019	0.121
Depth of invasion	4	405	FEM	0.823	0.402–1.684	0.594	0.267
Lymph node status	6	561	FEM	0.709	0.462–1.087	0.115	0.646
Metastasis	5	628	REM	0.492	0.251–0.965	0.039	0.022

REM, random-effects model; FEM, fixed-effects model; OR, odds ratio; CI, confidence interval.

There was no significant association between *β-catenin* overexpression in the cytoplasm and Dukes’ stages and lymph node status. The combined ORs were 0.87 (95% CI: 0.45–1.69, Z = 0.41, P = 0.685) and 0.78 (95% CI: 0.4–1.52, Z = 0.72, P = 0.469).

### Publication Bias

Egger’s test indicated that there was no evidence of significant publication bias after assessing the funnel plot (**Figure S1, S2, S3, S4**) for the studies included in our meta-analysis.

## Discussion

Approximately 60–80% of CRCs develop on the basis of an aberrant activation of the Wnt signaling pathway in which *β-catenin* serves as a central hub [34], [35]. There are many reports about the prognostic significance of *β-catenin* in CRC [20], [24], [28], [29]. Surprisingly, correlations between an immunohistochemically detected expression of *β-catenin* in CRC and prognosis are highly variable and contradictory. Thus, a quantitative meta-analysis that systematically determines the association of *β-catenin* expression with CRC survival was warranted. Our analysis indicated that *β-catenin* overexpression in the nucleus was significantly associated with progress disease and worse prognosis of CRC.

*β-catenin*, a central molecule of the Wnt signaling pathway, is expressed in epithelial cells in three main forms: membrane, cytoplasm and nucleus localization. When it is located in the membrane, it is responsible for cell-to-cell adhesion through forming complexes with E-cadherin and actin filaments. The other two forms are mainly involved in regulation of the Wnt signaling pathway. Cytoplasmic *β-catenin* is usually degraded upon interaction with the destruction complex formed by the three proteins APC, Axin and GSK3β and maintained at a low level in the absence of a Wnt ligand. Once a Wnt ligand engages with receptors, Axin translocates to the transmembrane receptor complex, thereby inhibiting the destruction complex. Consequently, *β-catenin* accumulates in the cell and undergoes translocation to the nucleus, where it activates specific Wnt target genes in conjunction with the T-cell factor/lymphoid enhancer factor (TCF/LEF) family of transcription factors. Under physiological conditions, Wnt activity is crucial for intestinal stem cells and crypt homeostasis. However, Wnt signaling also plays a key role in CCICs maintenance, which is the origin of tumor progression, therapy resistance and disease relapse.

It has been shown that migrating cancer stem cells (MCSCs), which play a crucial role in the metastasis of CRC, usually undergo nuclear *β-catenin* accumulation, cell-cycle arrest and epithelial–mesenchymal transitions (EMT). Notably, nuclear *β-catenin* was predominantly observed in the invasive front of CRC tissue. These observations concur with our finding that *β-catenin* overexpression in the nucleus was significantly associated with metastasis and worse prognosis of CRC. Some studies have also shown that the accumulation of *β-catenin* in the cytoplasm and nuclear translocation were inseparable processes and that some mechanisms were involved in the nuclear cytoplasmic shuttling of *β-catenin* [36]–[38]. In this meta-analysis, we analyzed all the eligible studies with *β-catenin* cytoplasmic data and found that *β-catenin* overexpression in the nucleus, rather than in the cytoplasm, influenced the survival of CRC patients. In fact, some investigators have argued that overexpression of *β-catenin* in the nucleus, rather than in the cytoplasm, might reflect *β-catenin* transactivating activity [39]. Some researchers have also suggested that increased cytoplasmic expression of *β-catenin* was not accompanied by nuclear accumulation [40]. This result may partially account for why the pooled results indicated that *β-catenin* overexpression in the cytoplasm had no relationship with the prognosis of CRC.

In this meta-analysis, we dealt with highly significant heterogeneity between the 18 studies. This heterogeneity could potentially affect the meta-analysis results. We only included studies that used immunohistochemistry to reduce heterogeneity as much as possible. However, the source and dilution of primary antibodies, evaluation standards, study location and adjuvant therapy conditions were quite different across studies, creating significant heterogeneity. Accordingly, we used random effects models to analyze the data, but the models did not identify the source of heterogeneity. To clarify the source of heterogeneity in this study, we performed stratified analysis according to study location, source of primary antibodies, evaluation standards and adjuvant therapy condition. When the analysis of OS was performed without consideration of these other factors, heterogeneity was detected (I^2^ 71.5% P = 0.000). When the analysis was limited to studies of Asia, heterogeneity was not detected (I^2^ 0% P = 0.414). Heterogeneity was also not detected (I^2^ 0% P = 0.45) when the analysis was limited to studies that defined *β-catenin* positive using a percentage. However, when the analysis of OS was limited to studies with primary antibodies from the same company and with adjuvant therapy, heterogeneity still existed (I^2^ 79.6% P = 0.008 and I^2^ 56.4% P = 0.076). This finding suggested that primary antibodies and adjuvant therapy did not contribute to heterogeneity in the results. These results suggest that the heterogeneity in this study could be partially explained by patient ethnicity and evaluation standards. Although meta-analysis is robust, certain limitations exist in this study. First, the study included in our meta- analysis was restricted only to articles published in English, which probably provided additional bias. Second, methodological differences of immunohistochemistry may contribute to heterogeneity. We could not perform subgroup analysis to explore this influence because few studies offered the concrete data. Third, HRs calculated from data or extrapolated from survival curves might be less reliable than direct analysis of variance.

In this study, we showed that *β-catenin* overexpression in the nucleus was significantly correlated with progress disease and a worse prognosis of CRC. Large, well-designed prospective studies are required to investigate the precise prognostic significance of *β-catenin* overexpression in the nucleus.

## Supporting Information

Figure S1 **Funnel plot to assess publication bias.** Egger’s publication bias plot showed no publication bias for studies regarding *β-catenin* expression in the nucleus and disease free survival (DFS) in the meta-analysis: the relationship between the effect size of individual studies (HR, vertical axis) and the precision of the study estimate (standard error, horizontal axis).
(TIF)

Figure S2 **Funnel plot to assess publication bias.** Egger’s publication bias plot showed no publication bias for studies regarding *β-catenin* expression in the nucleus and overall survival (OS) in the meta-analysis.
(TIF)

Figure S3 **Funnel plot to assess publication bias.** Egger’s publication bias plot showed the presence of publication bias for studies regarding *β-catenin* expression in the nucleus and Dukes’ stages in the meta-analysis.
(TIF)

Figure S4 **Funnel plot to assess publication bias.** Egger’s publication bias plot showed no publication bias for studies regarding *β-catenin* expression in the nucleus and metastasis in the meta-analysis.
(TIF)

Table S1 **PRISMA 2009 checklist.**
(DOC)
