# Supplementary figures and images for: β-catenin Overexpression in the Nucleus Predicts Progress Disease and Unfavourable Survival in Colorectal Cancer: A Meta-Analysis
Source: PLoS One. 2013 May 24;8(5):e63854. doi: 10.1371/journal.pone.0063854 (PMC3663842; doi:10.1371/journal.pone.0063854)

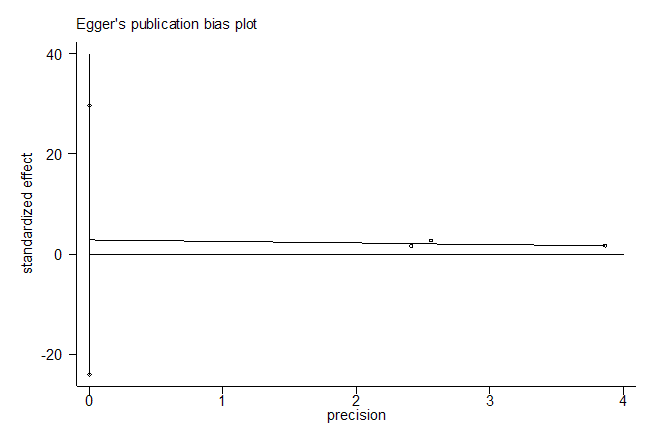

Supplement: Figure S1 — Funnel plot to assess publication bias. Egger’s publication bias plot showed no publication bias for studies regarding β-catenin expression in the nucleus and disease free survival (DFS) in the meta-analysis: the relationship between the effect size of individual studies (HR, vertical axis) and the precision of the study estimate (standard error, horizontal axis). (TIF) [file pone.0063854.s001.tif]

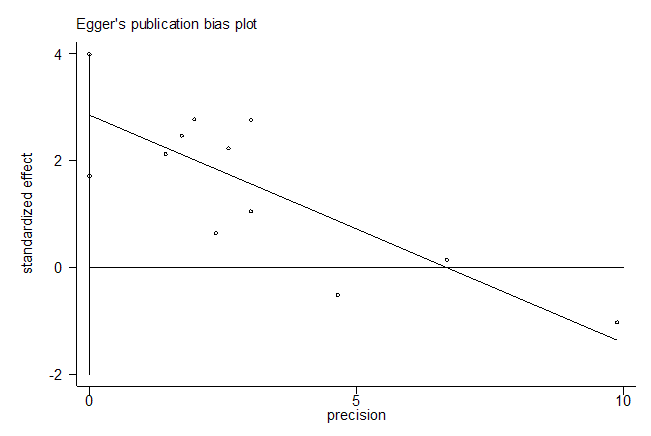

Supplement: Figure S2 — Funnel plot to assess publication bias. Egger’s publication bias plot showed no publication bias for studies regarding β-catenin expression in the nucleus and overall survival (OS) in the meta-analysis. (TIF) [file pone.0063854.s002.tif]

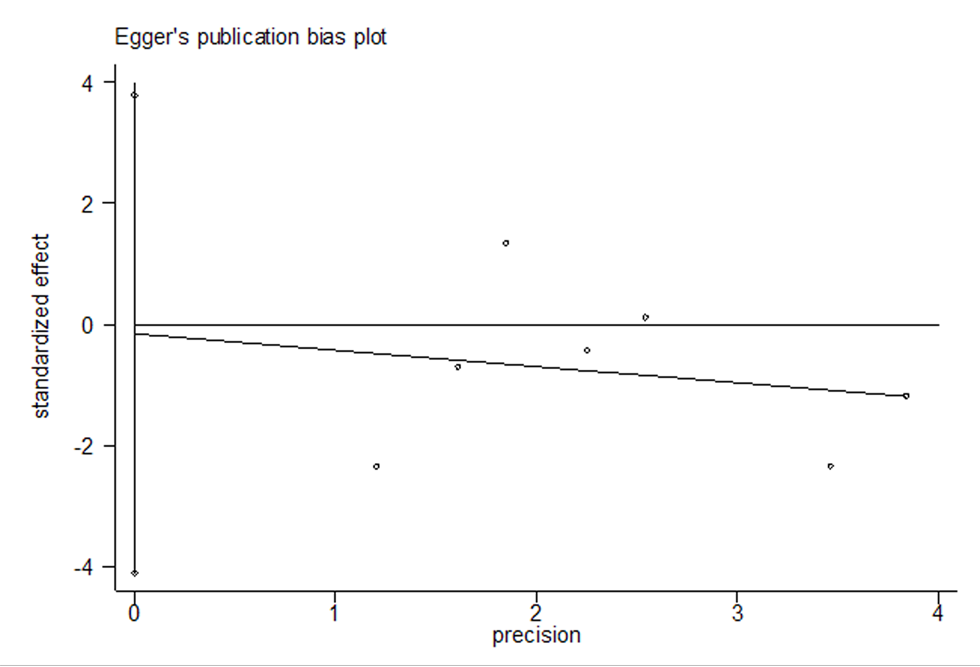

Supplement: Figure S3 — Funnel plot to assess publication bias. Egger’s publication bias plot showed the presence of publication bias for studies regarding β-catenin expression in the nucleus and Dukes’ stages in the meta-analysis. (TIF) [file pone.0063854.s003.tif]

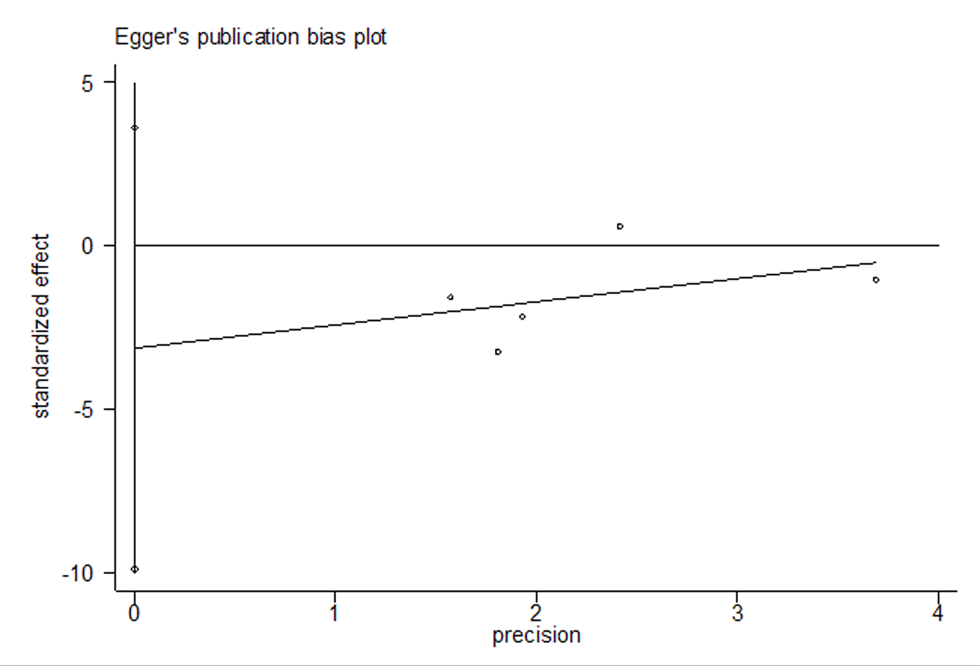

Supplement: Figure S4 — Funnel plot to assess publication bias. Egger’s publication bias plot showed no publication bias for studies regarding β-catenin expression in the nucleus and metastasis in the meta-analysis. (TIF) [file pone.0063854.s004.tif]
